# On Eruptions (Not Corymbiform) Produced by Nerve Influence

**Published:** 1877-03

**Authors:** 

**Affiliations:** London Hospital


ON ERUPTIONS (NOT CORYMBIFORM) PRODUCED BY NERVE INFLUENCE—*By Mr. Hutchinson, London Hospital.*—In some former remarks I asked your attention to the importance of the symptom of corymbiform arrangement of the spots in the cases of skin eruptions, as an indication of their location by the nerves of the part. This, however, is only one division of the class of skin disorders which are excited through the nervous system. I now wish to bring before you certain other disturbances in the nutrition of the skin, and especially of its glands, which occur not in connection with changes in or irritation of certain single nerve-trunks (or their nuclei), but which are produced by a general change—let us say in nerve-tone—which, induced by many and various causes, has at once for its result the production of very definite and peculiar results.

I will take, first, the common malady known as acne. In this disease the sebaceous glands of certain special parts inflame and show a tendency, more or less slowly, to suppurate. Closely allied to acne—indeed, often only local varieties of it, or great exaggerations—are styes on the eyelids, ecthyma, and boils. Now, let me assert that all these forms of skin disease can be proved in many instances to have far more to do with disturbance of nerve-tone than with either blood-poisoning on the one hand, or blood-impoverishment on the other. Blood changes may be, and usually are, to some degree present; but, let me repeat, the local acne-spot is due not to it, but to the nervous system. Do not allow the apparent irregularity of results to mislead you. Many coincident conditions are, no doubt, necessary to the occurrence of visible results, and in unraveling the cases we must take cognizance of all; but try carefully to assign to each its special share. We will then admit, *in limine*, that by no means in all persons can nerve disturbance excite acne. A certain favoring state of body is undoubtedly essential. You cannot fire off an empty gun, however good your percussion-cap. A person to be predisposed to acne must have a more or less coarse skin, with large sebaceous glands.—*Clinic.*

---

Bromide of Arsenic in the Treatment of Epilepsy.—Dr. Clemens, of Frankfort-on-the-Maine, has employed bromide of arsenic for twenty years in the treatment of diseases of the nervous system, and especially of epilepsy, and claims that he has obtained astonishing results with it. He uses the liquor arsenic. brom., and gives one or two drops in a glass of water, once, or, if necessary, twice daily. These minute doses may be given for months, or even years, without producing the usual unpleasant effects of a long-continued arsenical course. All his cases of epilepsy have been markedly relieved and improved by this remedy, but in two cases only has it produced a complete cure. In many cases of incurable epilepsy, complicated with idiocy and deformities of the skull, the fits were reduced in number from twenty, in the twenty-four hours, to four, or even two—a result that has been obtained by no other treatment. In connection with the bromide of arsenic, an almost exclusively meat diet is advised. The patients should be as much as possible in the open air in the day time, and their windows should be kept open at night. Unlike bromide of potassium, this remedy does not require to be given in increasing doses, and instead of interfering with digestion, improves the nutrition and strength. Dr. Clemens has employed the following formula since 1859, and thinks that it ought to replace Fowler's Solution, which is irrational in its composition and uncertain in its action. This solution becomes stronger with time, the chemical union of the bromide with the arsenite of potash becoming more and more perfect: ℞.—pulv. arsenic. alb. potass. carb. e. tartar., a a ʒj.; coque cum aqua distil. lb.ss. ad solut. perfect., adde, aq. evaporat. restituta, aquæ destil. ℥xij. dein adde brom. pur. ʒij. refrigerat. stet. per sufficient. temp. ad decol., S. liq. arsenic. bromat.—*The Medical Record*, September 9, 1876.

